# CHOP-mediated IL-23 overexpression does not drive colitis in experimental spondyloarthritis

**DOI:** 10.1038/s41598-024-62940-0

**Published:** 2024-05-29

**Authors:** Fatemeh Navid, Tejpal Gill, Lilah Fones, Jules D. Allbritton-King, Kelly Zhou, Isabel Shen, Jinny Van Doorn, Francesca LiCausi, Antony Cougnoux, Davide Randazzo, Stephen R. Brooks, Robert A. Colbert

**Affiliations:** 1 Pediatric Translational Research Branch, NIAMS, NIH, Bethesda, MD 20892 USA; 2 Clinical and Investigative Orthopedics Surgery Unit, NIAMS, NIH, Bethesda, MD 20892 USA; 3 Section on Molecular Dysmorphology, NICHD, NIH, Bethesda, MD 20892 USA; 4 Light Imaging Section, NIAMS, NIH, Bethesda, MD 20892 USA; 5 Biodata Mining and Discovery Section, NIAMS, NIH, Bethesda, MD 20892 USA

**Keywords:** Cytokines, Protein folding, Ankylosing spondylitis

## Abstract

HLA-B27 is a major risk factor for spondyloarthritis (SpA), yet the underlying mechanisms remain unclear. HLA-B27 misfolding-induced IL-23, which is mediated by endoplasmic reticulum (ER) stress has been hypothesized to drive SpA pathogenesis. Expression of HLA-B27 and human β_2_m (hβ_2_m) in rats (HLA-B27-Tg) recapitulates key SpA features including gut inflammation. Here we determined whether deleting the transcription factor CHOP (*Ddit3−*/−), which mediates ER-stress induced IL-23, affects gut inflammation in HLA-B27-Tg animals. ER stress-mediated *Il23a* overexpression was abolished in CHOP-deficient macrophages. Although CHOP-deficiency also reduced *Il23a* expression in immune cells isolated from the colon of B27+ rats, *Il17a* levels were not affected, and gut inflammation was not reduced. Rather, transcriptome analysis revealed increased expression of pro-inflammatory genes, including *Il1a*, *Ifng* and *Tnf* in HLA-B27-Tg colon tissue in the absence of CHOP, which was accompanied by higher histological Z-scores. RNAScope localized *Il17a* mRNA to the lamina propria of the HLA-B27-Tg rats and revealed similar co-localization with *Cd3e* (CD3) in the presence and absence of CHOP. This demonstrates that CHOP-deficiency does not improve, but rather exacerbates gut inflammation in HLA-B27-Tg rats, indicating that HLA-B27 is not promoting gut disease through ER stress-induced IL-23. Hence, CHOP may protect rats from more severe HLA-B27-induced gut inflammation.

## Introduction

Spondyloarthritis (SpA) is an immune-mediated inflammatory disease characterized by multiple sites of inflammation including the gastrointestinal tract, entheses and joints in the axial and peripheral skeleton, and eyes^1^. Up to 60% of patients with ankylosing spondylitis (AS), the prototype of SpA, have subclinical gut inflammation, with up to 10% developing overt inflammatory bowel disease (IBD)^2^. Genome wide associated studies suggest a shared genetic predisposition between SpA and IBD patients, supporting the importance of a “gut-joint-axis” in SpA^2^. It is unkown whether the same mechanisms are operative at different sites of inflammation. Therefore, understanding mechanisms leading to gut inflammation may clarify our overall understanding of pathogenesis and facilitate the development of better therapeutic strategies to treat different clinical features of SpA.

The major histocompatibility complex (MHC)-encoded class I molecule HLA-B27 is the single most important genetic risk factor predisposing to SpA, with over 80% of AS patients carrying this HLA-B allele^1^. Expression of HLA-B27 and human β_2_-microglobulin (hβ_2_m) in rats (HLA-B27-Tg) can generate gut and joint inflammation, two key features of human spondyloarthritis, and thus these animals provide a model to better understand pathogenic mechanisms^3^. In HLA-B27-Tg rats, CD8+ T cells are not required for disease^4,5^, which instead appears to be a consequence of CD4+ Th17 T cells that accumulate in inflammatory lesions and produce IL-17A and related cytokines^6,7^. This model has contributed to the development of effective therapeutic approaches such as biologics blocking IL-17A in humans^8,9^. Expanded clones of CD8+ T cells recognizing HLA-B27 have now been found in humans with SpA, and thus a role for CD8+ T cells in human disease remains a possibility^10,11^.

Myeloid cell abnormalities resulting from HLA-B27 expression have been hypothesized to play a role in promoting CD4+ Th17 T cell expansion and activation^12,13^. The propensity of HLA-B27 to misfold may underly some of these abnormalities. For example, HLA-B27 heavy chains form disulfide-linked dimers in the endoplasmic reticulum (ER) (misfold)^14^, which can prevent efficient ER-associated degradation (ERAD) and contribute to the accumulation of HLA-B27^13^ and activation of the unfolded protein response (UPR)^15^.

The UPR contributes to increased expression of IL-23 by upregulating *Il23a*/IL-23p19^6^ in immune cells through upregulation of C/EBP homologous protein (CHOP, also known as growth arrest and DNA damage-inducible protein 153 [GADD153])^16^. Encoded by *DDIT3*, CHOP is a transcription factor that acts as both an activator and repressor^17^. CHOP expression is induced during ER stress in a wide variety of cells including macrophages^17^, through the initial activation of protein kinase RNA-like endoplasmic reticulum kinase (PERK)^18^. Activated PERK phosphorylates eukaryotic translation initiation factor 2α (eIF2α), which leads to preferential translation of the transcription factor ATF4, that converges on the promoters of target genes, including CHOP^19–21^. UPR-induced CHOP binds directly to the *Il23a* promoter and augments gene expression as shown in monocyte-derived dendritic cells after exposure to LPS and thapsigargin, a chemical inducer of the UPR^16^. IL-23 acts as an upstream modulator of the T lymphocytes driving Th17 expansion and production of IL-17 and related cytokines that contribute to the pathogenesis of inflammatory diseases such as SpA^22^.

Here, we asked whether CHOP plays an important role in HLA-B27-mediated gut inflammation in experimental SpA by promoting activation of the IL-23/IL-17 axis. Surprisingly, we found that CHOP is not required for this component of the SpA phenotype. Despite reducing UPR-mediated increases in *Il23a* expression in myeloid cells, eliminating CHOP had no significant effect on CD3+ T cell accumulation and *Il17a* expression in gut tissue. Rather, our results suggest that CHOP may actually protect HLA-B27-Tg rats from more severe inflammation and increased expression of other pro-inflammatory cytokines like TNF, IFNγ, and IL1α.

## Materials and methods

### Animals

Hemizygous HLA-B*27:05 and human β_2_m-transgenic (HLA-B27-Tg) Lewis rats carrying 55 copies of HLA-B27 and 66 copies of human β_2_m in the 33–3 locus were used for this study^23^. *Ddit3−*/*−* rats (CHOP deficient) were generated on the Lewis background via CRISPR/Cas9 editing (Transposagen now Hera BioLabs, Lexington, KY) using the following CRISPR target site: Crispr-1 GGAGCTGGAAGCCTGGTAT and Crispr-2 GGTGCCCCCAATTTCATCTG. This created a 25 bp deletion in exon 1 of *Ddit3* resulting in a translational frameshift and the appearance of downstream in-frame stop codons (see Suppl. Fig. 1). Animals carrying two copies of this deletion, designated as *Ddit3−*/−, were bred with hemizygous HLA-B27-Tg Lewis rats to generate the cohorts used for these studies. We refer to *Ddit3*−/− rats as CHOP-, and all experiments assessing the role of CHOP in HLA-B27-Tg rats were performed by comparing CHOP+ (*Ddit3*+/+) to CHOP− (*Ddit3*−/−) rats with abbreviated names as shown in Suppl. Table 1. Rats were bred and housed in Association for Assessment and Accreditation of Laboratory Animal Care-approved facilities on the Bethesda campus of the NIH. All animal experiments were approved by the Animal Care and Use Committee at the National Institute of Arthritis and Musculoskeletal and Skin Disease. Rats were euthanized with compressed carbon dioxide (CO2) gas inhalation according to NIH ARAC Guidelines. Death was verified by ascertaining cardiac/respiratory arrest or noting fixed and dilated pupils, followed by cervical dislocation or double pneumothorax. All methods reported in this study are in accordance with the relevant guidelines and regulations and follow the ARRIVE guidelines.

### Histologic evaluation

Paraffin-embedded colon tissue (distal ~ 2 cm) was hematoxylin and eosin (H&E)-stained and scored with modification as described previously^24^ by observers blinded to genotype. Key components of the scoring system including gut-associated lymphoid tissue (GALT)/severity score (0 or 1), goblet cell loss (0–3), immune cell infiltrate (0–3), and area of tissue affected (0–3) were scored and then summed to obtain a final histology score. Thus, total scores could range from 0 to 10.

Histology Z-scores were calculated using the following formula:

*z* = (*x* − *μ*)*/*σ; where z = Z-score; x = the observed value; μ = the mean of the corresponding B27- control group; σ = the standard deviation of the corresponding B27- control group.

### *Colon* tissue transcriptome analysis

Colon tissue samples obtained from 2–3- and 6–7-month-old rats (female and male) were homogenized in TRIzol reagent (Thermo Fisher Scientific). RNA was isolated using a standard phenol–chloroform protocol. RNA quantity and quality was assessed (Agilent) and RNA with an integrity number > 8 was used for RNASeq analysis. Libraries were prepared according to the manufacturers guide (Illumina). The Illumina Novaseq 6000 system was used to perform 50-base, single-end sequencing. Raw data were mapped to a custom genome based on rat rn6 but incorporating HLA-B27 and hβ_2_m. Partek Genomics Suite 7.0 was used to calculate Reads per Kilobase Million (RPKM) and to analyze differential gene expression by ANOVA. For further analysis, only genes where at least one sample had an RPKM > 1, and a minimum of twofold change, and p < 0.05 were used. Partek Genomics 7.0 was also used for principal component analysis (PCA), hierarchical clustering/global heat map and for Volcano plot analysis.

### Isolation of bone marrow-derived macrophages (BMM)

Bone marrow was obtained from the tibias and femurs of 8–12 weeks old rats following euthanasia. Nonadherent bone marrow monocytes were incubated with M-CSF (Peprotech) as described previously to drive BMM formation^25^. Cells were used for experiments at day 7.

### Isolation of immune cells from *colon* tissue

Immune cells were isolated from colon tissue as described previously with some modifications^26^. Briefly, after removing connective tissue, colon tissue was washed in PBS several times, and then cut into 0.5 cm sections in Hank’s balanced salt solution (HBSS, Ca^2+^/Mg^2+^, Thermo Fisher Scientific) supplemented with 5% FBS and 1% Antibiotic–Antimycotic (Thermo Fisher Scientific). To detach mucosa from the epithelial layer, sections were incubated in 1 mM DTT in HBSS supplemented with 5% FBS for 20 min at 37 °C with shaking. Supernatants were removed and tissues were vortexed briefly and then placed in HBSS containing 300 U/mL collagenase-II (Millipore-Sigma) for 40 min at 37 °C with shaking. Supernatants were centrifuged to pellet cells, followed by gradient centrifugation with Lympholyte (Cedarlane) to collect the mononuclear fraction. Cells were then lysed in TRIzol reagent (Thermo Fisher Scientific) and RNA was isolated using Direct-zol RNA Miniprep plus kit (Zymo Research).

### Reagents and antibodies

The following antibodies were used for immunoblotting: mouse anti-CHOP antibody (Cell Signaling Technology, #2895), rabbit anti-Lamin B1 (Abcam, #ab16048) and mouse anti-GAPDH (Santa Cruz Biotechnologies, #sc-32233). As secondary antibodies, horseradish peroxidase (HRP)-conjugated anti-rabbit and anti-mouse (R&D Systems, #HAF008, #HAF007) were used. For immunofluorescence, mouse anti-CHOP antibody (Cell Signaling Technology, #2895) and Alexa Fluor 594 or 488 conjugated donkey anti-mouse secondary antibody (Life Technologies, #A21203, #A21202) were used.

### Immunofluorescence

For immunofluorescence analysis, BMM were fixed with 4% formaldehyde for 15 min at room temperature. After washing with PBS, cells were incubated with 5% normal goat serum (Life Technologies) in PBS containing 0.3% Triton X-100 (Americanbio) for one hour at room temperature, followed by overnight incubation at 4 °C with the primary anti-CHOP antibody (Cell Signaling Technology, #2895). After washing with PBS, cells were incubated with a fluorescent-labeled secondary antibody in the dark for one hour at room temperature. The nucleus was visualized with DAPI (Sigma-Aldrich). Immunofluorescence was evaluated by using the Leica DMI4000 B Fluorescence Research inverted microscope (Leica Microsystems) at 20× magnification.

### Protein separation and immunoblotting

To evaluate CHOP expression, nuclear and cytosolic fractions were prepared from BMM using NE-PER Nuclear and Cytoplasmic Extraction Reagents (Thermo Fisher Scientific). Proteinase inhibitor cocktail tablets (EDTA-free) (Roche) were added to the buffers. For immunoblotting, cytoplasmic and nuclear fractions were diluted with 5× sample buffer (Thermo Fisher Scientific) and 10× reducing agent (Thermo Fisher Scientific), then incubated in boiling water for 5 min. Proteins were separated on 4–20% Tris/Glycine gels (BioRad) and immunoblotting was performed as described^32^. The following primary antibodies were used: mouse anti-CHOP (Cell Signaling Technology, #2895), rabbit anti-Lamin B1 (Abcam, #ab16048), and mouse anti-GAPDH (Santa Cruz Biotechnologies, #sc-32233). HRP-conjugated secondary antibodies (R&D Systems, #HAF008, #HAF007) against their respective primary antibodies were incubated with membranes for 1 h at room temperature. Proteins were visualized using the Pierce ECL Western Blotting Substrate (Thermo Fisher Scientific).

### Gene expression analysis

Total RNA was reverse transcribed according to the manufacturer’s guidelines using an iScript cDNA Synthesis kit (BioRAD). To determine relative gene expression, quantitative PCR (qPCR) was performed using a commercially available Taqman Assay (Thermo Fisher Scientific) specific for rat *Il23*α (Rn00590334_g1), rat *Il17a* (Rn01757168_m1) and rat *Actb* (Rn00667869_m1) or rat *Ppia* (Rn00690933). The qPCR was performed using an iCycler Thermo Cycler (BioRad). For calculating the 2^−ΔΔCT^ the CT values measured for B27+ CHOP+ and B27+ CHOP− were compared to their respective CT values for B27- controls.

### RNAScope analysis

Fluorescent in situ hybridization was performed on formalin-fixed, paraffin-embedded colon tissue using the RNAscope Multiplex Fluorescent V2 Assay kit (ACD) according to the manufacturer’s instructions. *Il17a* (Cat. No. 313611-C3) and *Cd3e* (Cat. No. 409781) were visualized. Positive and negative controls were obtained using control probes provided by the manufacturer (Cat. No. 321821 and 321,831, respectively). Briefly, slides were incubated at 60 °C for 60 min then submerged in Xylene twice for 5 min, followed by pure ethanol twice for 2 min. After drawing a hydrophobic barrier using an ImmEdge pen (Vector Laboratories), slides were incubated for 10 min at room temperature in Hydrogen Peroxide Pretreatment solution, rinsed for 2 min in distilled/de-ionized water (DI H_2_O), incubated for 30 min at 40 °C in ACD Custom Pretreatment solution, and then rinsed again in DI H_2_O twice for 2 min each. Slides were then incubated at 40 °C for 2 h in the appropriate target or control probe solution, and after washing stored at room temperature in 5 × SSC buffer. The following day, slides were incubated at 40 °C in Amp 1 solution for 30 min, Amp 2 solution for 30 min, and Amp 3 solution for 15 min, rinsing for twice for 2 min in 1 × Wash buffer between each incubation. Following amplification, the C1 target or control probe was developed by incubating at 40 °C in Multiplex HRP-C1 for 15 min, with 1:1500 Opal 570 dye (Akoya Biosciences) in TSA Buffer for 30 min, and HRP Blocker for 15 min, rinsing twice for 2 min in 1 × Wash Buffer between each incubation. This step was repeated for the C3 target or control probes using Opal 620 dye (Akoya Biosciences). Finally, slides were incubated for 30 s at room temperature in DAPI solution and immediately mounted using (Vector Laboratories).

### Confocal microscopy

Micrographs were acquired on a Leica TCS SP8 X confocal system driven by the LAS X software (Leica) employing a Plan Apochromatic CS2 40X/1.3NA oil immersion lens (Leica) with pinhole size set to 1.0 Airy Units (AU). Excitation of fluorophores was achieved through a solid state 405 nm laser (for DAPI) and a tunable White Light Laser (Leica) with wavelengths set at 550 nm (for Opal 570) and 588 nm (for Opal 620). Micrographs were acquired employing a tile scanning approach to acquire large arears of the colon sections, using the Navigator module of the LAS X software. The number of tiles required to cover the tissue areas was automatically determined depending on the size of the area. After the scan, the tiles were stitched together to generate the images shown in Fig. 4A. Images were acquired in sequential scanning modality to avoid crosstalk between fluorescence channels and files were exported as .tiff with lossless compression. When needed, intensity levels were linearly adjusted post-acquisition. Analysis of colocalization between *Cd3e* and *Il17a* was performed calculating the Pearson’s correlation coefficient using Imaris 9.9 (Bitplane).

### Statistical analysis

Student’s *t* test in GraphPad Prism Version 6.0b (LaJolla, CA) was used to assess differences between means, with *p* < 0.05 considered statistically significant. For the histological scores and the qPCR analyses the non-parametric Kruskal–Wallis multiple comparisons test was used. For calculating the statistics of the 2^−ΔΔCT^ values, the Mann–Whitney test was used on the ΔCT values. For transcriptome analysis differentially gene expression was evaluated using ANOVA as described under this “Method” section above. Unless otherwise stated, the figures show three independent experiments.

## Results

### Lack of CHOP expression in *Ddit3−/−* animals

**Figure 1:**
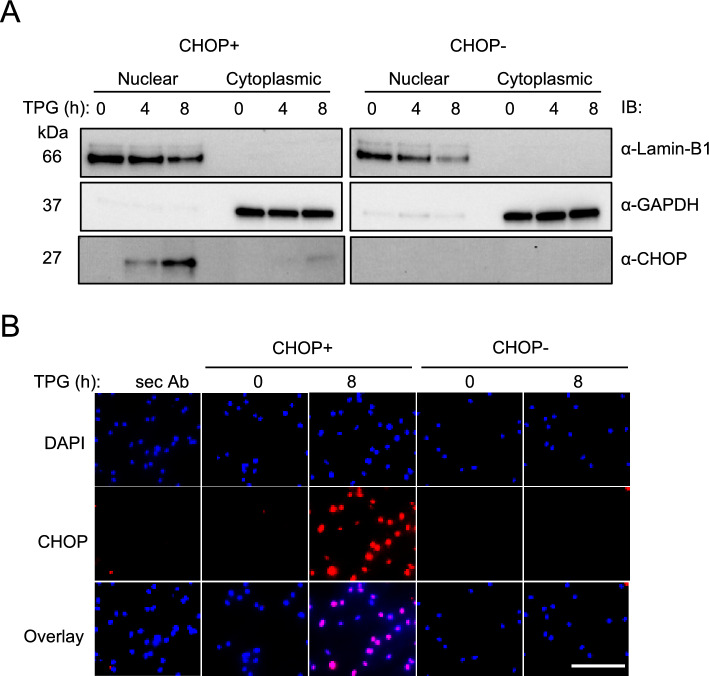
Lack of CHOP expression in *Ddit3−*/*−* rat macrophages upon UPR activation. (**A**) BMM from B27− CHOP+ (WT) and B27− CHOP− rats were generated and treated without or with 1 µM TPG for 4 or 8 h. Cells were lysed and nuclear and cytosolic fractions were obtained for Immunoblotting (IB) using antibodies against CHOP (α-CHOP) and as a loading control Lamin-B1 (α-Lamin-B1) or GAPDH (α-GAPDH). Images are representative of three independent experiments. Blots were cropped to highlight the region of interest; full blot images are provided in Suppl. Fig. 5. (**B**) BMM from B27+ CHOP+ and B27+ CHOP− rats were treated as described in (**A**). After fixation and permeabilization, cells were analyzed by immunofluorescence with anti-CHOP antibody (red). Nuclei are visualized by DAPI staining (blue). Immunofluorescence was evaluated using a Leica microscope at 20× magnification. Images are representative of three independent experiments. Scale bar, 50 μm.

To confirm that *Ddit3* editing eliminated CHOP expression, bone marrow macrophages (BMM) were generated from CHOP- (*Ddit3−/−*) and CHOP+ (*Ddit3*+*/*+) rats and examined with and without exposure to TPG, a chemical inducer of ER stress and UPR activation. Since CHOP can translocate rapidly to the nucleus after synthesis, we separated nuclear and cytoplasmic fractions for immunoblotting (Fig. 1A) and co-stained fixed and permeabilized cells with DAPI (blue) and anti-CHOP antibody (red) (Fig. 1B). CHOP is not detected in non-stressed wild type BMM, nor is it detected in CHOP− cells after TPG treatment (Fig. 1A,B). When detected, CHOP localizes mainly to the nuclear fraction of BMM on immunoblots (Fig. 1A) and co-localizes with DAPI on immunofluorescent images (Fig. 1B). These results confirm the successful deletion of CHOP expression in *Ddit3*−/− rats.

### CHOP deficiency eliminates increased *Il23a* expression during UPR activation

**Figure 2:**
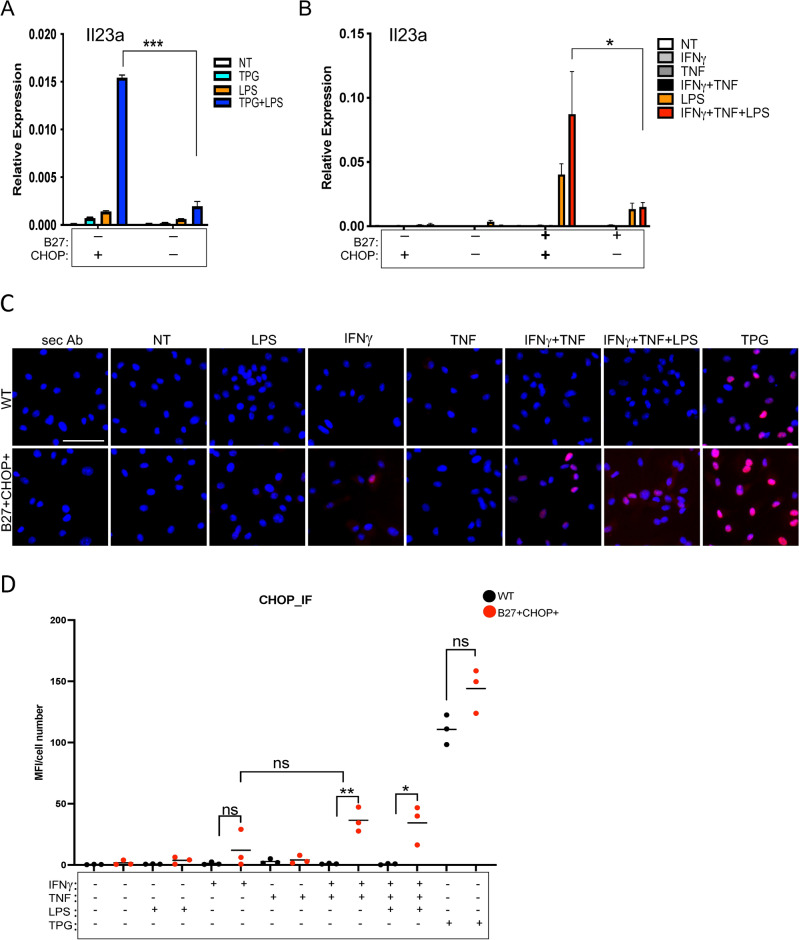
CHOP-deficiency reduces *Il23a* expression after UPR activation in LPS-treated rat macrophages. (**A**) BMM from B27− CHOP+ (WT) and B27− CHOP− rats were treated with TPG (1 µM) for 1 h. LPS (1 µg/mL) was added to TPG-treated and untreated cells and incubated for another 3 h. Cells were collected, RNA isolated, and *Il23a* expression analyzed by qPCR. *Ppia* expression was measured as a housekeeping gene. Quantitative data represent mean ± SEM of three experiments (**p < 0.01: ***p < 0.001) (**B**) BMM were treated with IFNγ (100 ng/mL), TNF (30 ng/mL) or IFNγ + TNF for 18 h, with LPS (1 µg/mL) added for another 6 h. Gene expression was analyzed by qPCR using primers for *Il23a* and *Ppia*. Results represent mean ± SEM of three experiments (*p < 0.05). (**C**) CHOP expression in B27+ CHOP+ macrophages compared to B27-CHOP + cells upon stimulation with IFNγ, TNF, IFNγ + TNF alone or in combination with LPS. After 18 h LPS (1 µg/mL) was added for another 3 h incubation. After fixation and permeabilization, cells were analyzed by immunofluorescence with anti-CHOP antibody (red). Nuclei are visualized by DAPI staining (blue). Immunofluorescence was evaluated using a Leica microscope (20× magnification). Images are representative of two independent experiments (scale bar, 250 μm). (**D**) Quantification of CHOP immunofluorescence signal of cells described in (**C**). For each condition three independent sections were analyzed for mean fluorescence intensity of CHOP and normalized to cell number. (*p < 0.05, **p < 0.01).

We next assessed whether CHOP deficiency affects synergistic *Il23a* induction that occurs when myeloid cells undergoing UPR activation are exposed to TLR agonists^6,13,16,22^. To test this, macrophages were treated with TPG for 1 h, followed by the addition of LPS for another 3 h. The synergistic induction of *Il23a* seen with LPS stimulation of TPG-treated cells is almost completely abrogated in CHOP-deficient macrophages (Fig. 2A), consistent with results from CHOP knockdown experiments in dendritic cells^16^. We then tested whether CHOP also mediates excess *Il23a* induction seen in HLA-B27-expressing cells when HLA-B27 is upregulated. For this experiment, BMMs were stimulated with IFNγ, TNF, or both cytokines (IFNγ + TNF) for 24 h, which upregulates HLA-B27 (Suppl. Fig. 2A–B, HLA-B27 recognized by HC10) and activates the UPR^6^ leading to an increase in CHOP expression with nuclear localization in HLA-B27+ cells upon cytokine stimulation, which is not observed in HLA-B27-negative cells expressing CHOP (B27− CHOP+) (or wild type; WT) (Fig. 2C–D). LPS was added for the final 6 h and *Il23a* expression was determined. Substantial *Il23a* upregulation by LPS is restricted to HLA-B27-expressing cells and is substantially reduced in the absence of CHOP (Fig. 2B). These results indicate that the increase in *Il23a* expression in HLA-B27-expressing cells in response to LPS is almost entirely CHOP dependent. However, it should also be emphasized that *Il23a* expression is not completely prevented in the absence of CHOP.

### CHOP deficiency exacerbates gut inflammation

**Figure 3:**
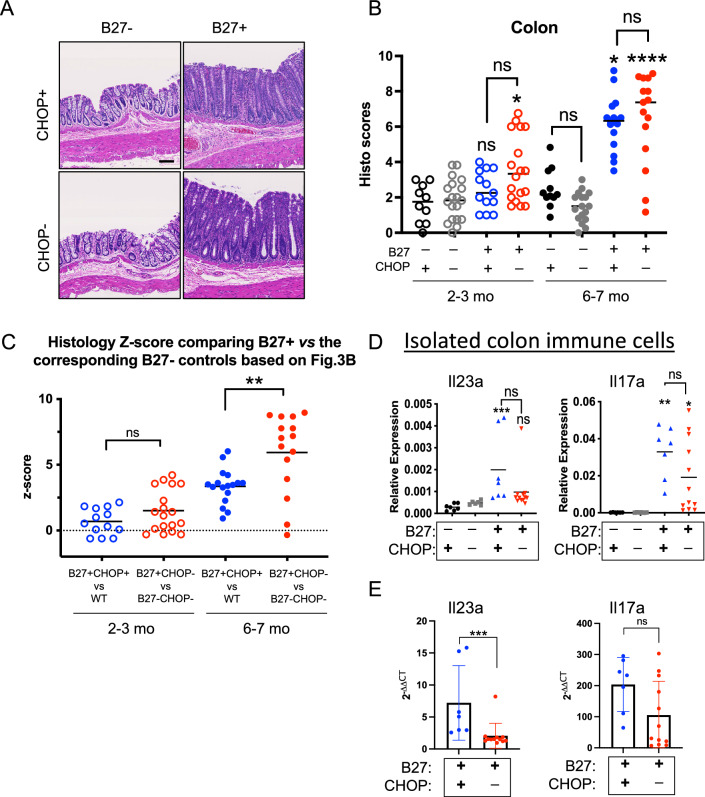
CHOP deficiency-mediated *Il23a* reduction does not prevent colon inflammation in HLA-B27-Tg rats. (**A**) Representative H&E staining of colon samples from 6–7 mo old B27− CHOP+ (WT), B27− CHOP−, B27+ CHOP+ and B27+ CHOP− rats (scale bar, 100 µm). (**B**) Histology scores from colon tissue of 2–3 and 6–7 mo old rats. Each data point represents the average score of 4–8 tissue sections from a single rat. Bar indicates the mean for all data points from a single genotype (*p < 0.05; ****p < 0.0001). The asterix on top of the B27+ CHOP+ (blue) or B27+ CHOP− (red) data points are showing the significance of these groups compared to their corresponding B27− control group (black or grey, respectively). (**C**) Histology Z-scores for B27+ and B27− rats in the presence and absence of CHOP. Histology scores for B27- rats in the presence of CHOP or the absence of CHOP were averaged for each age range. The average score was used as the denominator to calculate the Z-score for each B27+ rat of comparable CHOP genotype. Each data point represents a single rat. (**p < 0.01). The dotted line indicates the reference Z-score corresponding to B27− CHOP+ or B27− CHOP− controls. **D**) Immune cells isolated from 6–7 mo old rats were analyzed by qPCR for *Il23a* and *Il17a* expression normalized to *Actb*. **E**) The data are showing the relative gene expression foldchange of B27+ CHOP+ and B27+ CHOP− compared to their respective B27− controls calculated as 2^−ΔΔCT^ values from the data shown in (**D**). Each data point represents a single rat (*p < 0.05, **p < 0.01, ***p < 0.001). The asterix on top of the B27+ CHOP+ (blue) or B27+ CHOP− (red) data points are showing the significance of these groups compared to their corresponding B27− control group (black or grey, respectively).

Gut inflammation in susceptible HLA-B27-Tg rats begins at about 2-months of age with histology scores reaching a maximum at about 6 months of age^25^. Hence, the effect of CHOP-deficiency on gut inflammation was evaluated at 2–3 and 6–7 months of age by histological scoring. There was no apparent reduction in colon inflammation in the absence of CHOP in HLA-B27-Tg rats (Fig. 3A,B). However, we noted that CHOP-deficiency tends to reduce colon histology scores in HLA-B27 negative animals at 6–7 months of age compared to B27− CHOP+ (WT) rats, although the difference is not significant (Fig. 3B). It should be noted that this reduction in histology scores in the absence of CHOP is merely changing the baseline score, since HLA-B27 negative animals do not develop gut inflammation. In light of this difference in baseline scores, we generated histology Z-scores for HLA-B27+ to their respective HLA-B27− rats in the presence and absence of CHOP and found that inflammation was worse in HLA-B27+ animals in the absence of CHOP at 6–7 months of age compared to HLA-B27+ CHOP+ rats (Fig. 3C). The Z-score was calculated as described in Materials and Methods and provides a comparison of the individual histology scores of the B27+ group to the mean of their corresponding B27- control group taking into account the variability of the control group. This suggests that as inflammation progresses from 2–3 to 6–7 months of age, CHOP may actually protect HLA-B27+ rats from more severe gut inflammation. Additionally, we plotted the single categories of each histological score of the 6–7 month old animals to further dissect whether CHOP deficiency affects specific components of the score (Suppl Fig. 3). There were no statistically significant differences in the various components of the overall score, although one can appreciate that the major contributors to the increased scores in the absence of CHOP are fewer goblet cells, greater inflammatory infiltrates, and the area affected.

We then determined the relative expression of *Il23a* and *Il17a* in immune cells isolated from 6–7-month-old animals. As expected, there was an increase in *Il17a* expression seen in cells isolated from HLA-B27+ compared with HLA-B27− controls (Fig. 3D). But in contrast to B27+ CHOP+ cells, *Il23a* was not significantly increased in HLA-B27+ CHOP− rats, indicating the loss of CHOP-mediated *Il23a* overexpression (Fig. 3D). This loss of *Il23a* expression in B27+ CHOP− cells is further apparent, when foldchange using the 2^−ΔΔCT^ method was calculated, showing that there is significantly more *Il23a* produced in B27+ CHOP+ compared to B27+ CHOP− cells (Fig. 3E). While there was a tendency toward decreased *Il17a* expression in HLA-B27+ CHOP-deficient rats the difference was not statistically significant (Fig. 3D,E).

**Figure 4:**
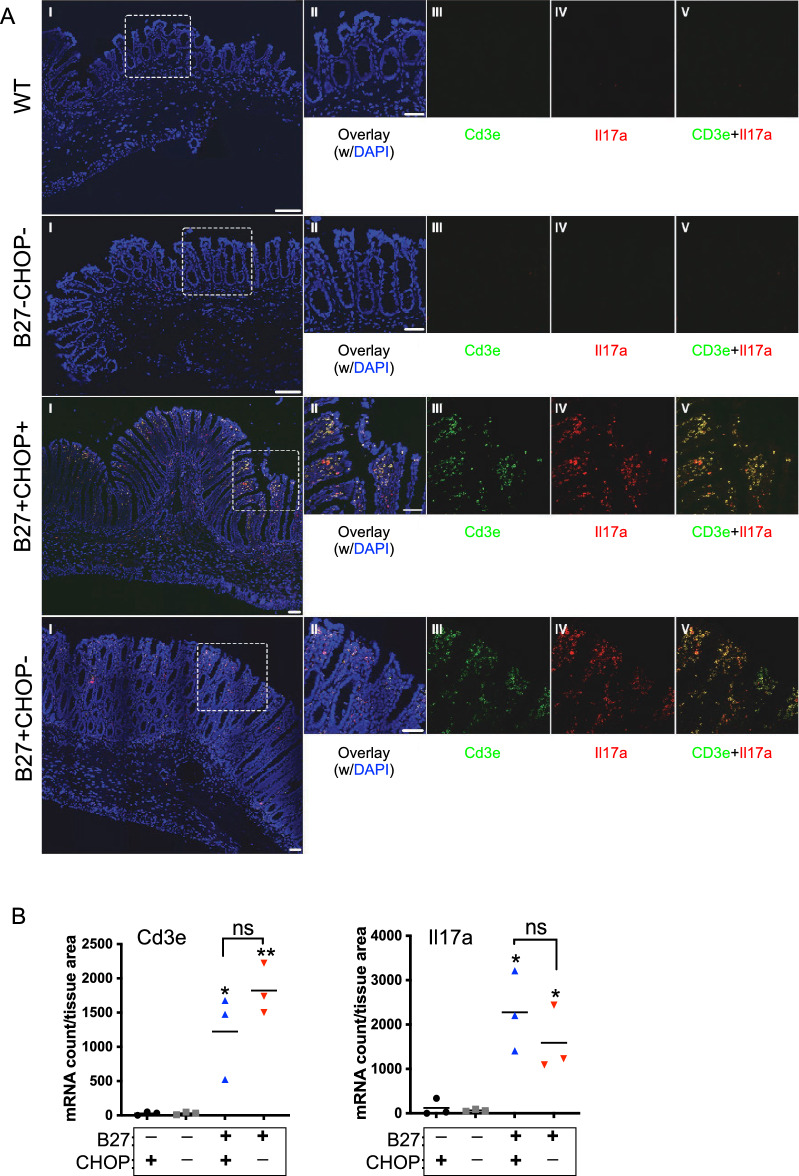
CHOP deficiency does not alter *Il17a* and *CD3e* expression in HLA-B27-Tg colon tissue. (**A**) Representative confocal micrographs of in situ hybridization of *Il17a* and *Cd3e* mRNAs of colon sections from 6–7 mo old rats. Image (I) represents a tile scan of a colon section with *Cd3e* mRNA (green), *Il17a* mRNA (red) and nuclei (DAPI, blue). Images II-V show magnified view of the dashed box in image I: overlay (II), *Cd3e* (III), *Il17a* (IV) and *Cd3e* + *Il17a* (V). Scale bars, 50 μm. (**B**) Quantification of *Il17a* and *Cd3e* mRNAs shown in (**A**) normalized on the area of lamina propria from three independent experiments (*p < 0.05, **p < 0.01). The asterix on top of the B27+ CHOP+ (blue) or B27+ CHOP− (red) data points are showing the significance of these groups compared to their corresponding B27− control group (black or grey, respectively).

Since CD3+/CD4+ Th17 T cells are expanded and activated in the colon of HLA-B27-Tg rats^6^, we performed in situ RNAScope analysis to localize CD3+ (*Cd3e*) and *Il17a*-expressing cells. Nuclei were visualized using DAPI and confocal images were analyzed. *Il17a* and *CD3e* mRNA signals were absent in non-inflamed tissue from WT (HLA-B27−) rats in the presence or absence of CHOP (Fig. 4A,B). In sections from HLA-B27-Tg rats, *Il17a* and *CD3e* mRNA staining localized mainly to the lamina propria (Fig. 4A), and semi-quantitative analysis of RNAScope images confirmed significant increases of *Il17a* and *Cd3e*-expressing cells in inflamed tissue of the HLA-B27-Tg animals (Fig. 4A,B). There was no significant reduction in *Il17a* mRNA in the absence of CHOP, nor was there any reduction in *Cd3e* (Fig. 4B). We then assessed co-localization of *Il17a* and *Cd3e* mRNAs from three experiments and found that they were similar in B27+ CHOP+ and B27+ CHOP− tissue (Pearson’s correlation 0.62 ± 0.17 vs. 0.59 ± 0.20, respectively). This suggests that the majority of IL-17A is produced by CD3+ cells, and that eliminating CHOP expression has no effect, and that there may be other cells expressing IL-17A in the inflamed colon of HLA-B27-Tg rats. For example, CD3- innate or adaptive lymphoid cells (e.g. γδT cells), natural killer cells, and/or neutrophils can express IL-17A^27^ with varying dependency on IL-23^28^. Taken together, these data indicate that excess *Il23a* related to HLA-B27-induced UPR and CHOP upregulation is not driving colitis in this model.

### CHOP deficiency increases pro-inflammatory cytokine expression in the colon of HLA-B27-Tg rats

**Figure 5:**
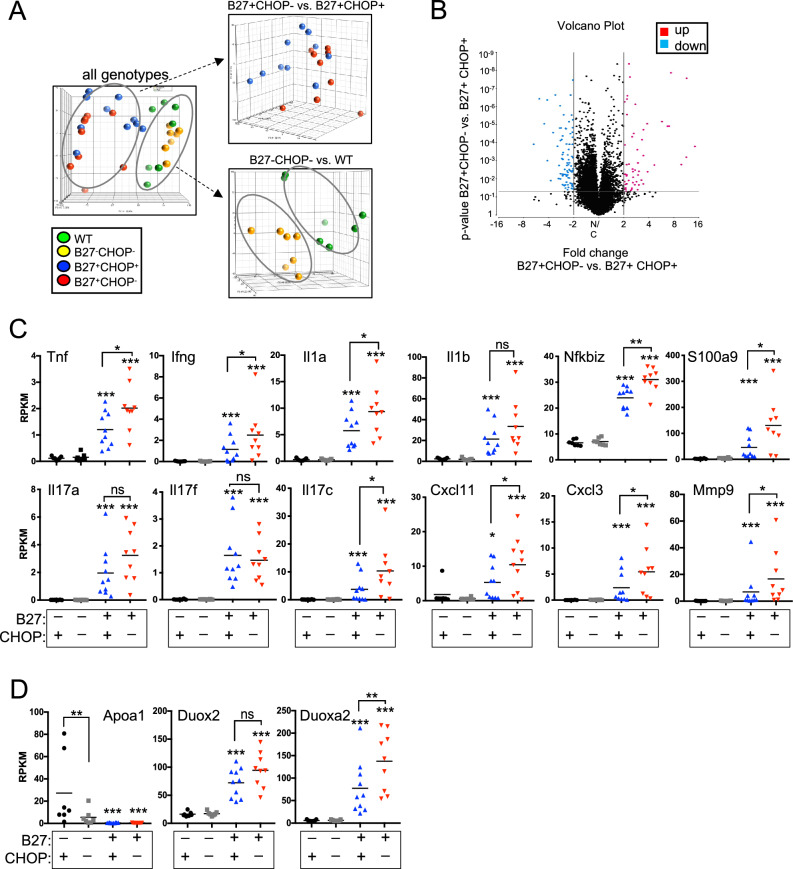
CHOP deficiency increases expression of pro-inflammatory genes in HLA-B27-Tg rat colon tissue. (**A**) Principal component analysis (PCA) of transcriptome using RNASeq results from 6–7 mo old rats: B27− CHOP+ (WT) (green), B27− CHOP− (yellow), B27+ CHOP+ (blue) and B27+ CHOP− (red) rats. Dotted lines indicate a PCA plot showing either the two B27- control genotypes (CHOP+ and CHOP-) or the two B27+ genotypes. Each dot represents a single animal. (**B**) Volcano plot of RNASeq data from colon tissue of 6–7 mo old B27+ CHOP− compared to B27+ CHOP+ animals. In red are the genes that are overexpressed (> twofold difference) (78 genes) and in blue the genes that are underexpressed (< twofold difference) (88 genes). The y-axis indicates the *p*-value and the x-axis the fold change. (**C**) Expression of selected genes in colon tissue. Individual genes represent key pro-inflammatory factors. The graphs are showing log_2_-transformed reads per kilobase million (RPKM) values for each gene. Each data point represents an individual rat with bars showing mean expression based on 7–10 rats per genotype. (**D**) Expression of additional genes *Apoa1*, *Duox2*, and *Duoxa2*, with data derived and analyzed as in (**C**). Results represent mean of 7–10 rats per genotype (*p < 0.05; **p < 0.01; ***p < 0.001). The asterix on top of the B27+ CHOP+ (blue) or B27+ CHOP− (red) data points are showing the significance of these groups compared to their corresponding B27− control group (black or grey, respectively).

To further assess the effects of CHOP-deficiency, we analyzed gene expression in colon tissue from 6–7-month-old animals using RNASeq. Principal Component Analysis of the overall transcriptome revealed clustering according to genotypes with the largest difference due to HLA-B27 expression (Fig. 5A). The presence or absence of CHOP also alters the transcriptome in both HLA-B27+ (Fig. 5A, top right; blue *vs.* red, respectively) and HLA-B27-negative (Fig. 5A, bottom right, green *vs.* yellow, respectively) rats. Genotype-specific differences are also apparent when gene expression is analyzed by unsupervised clustering as showed in a heat map (Suppl. Fig. 4A). Gene expression differences that are due to the absence of CHOP in HLA-B27+ animals are shown in the volcano plot (Fig. 5B), with 78 genes significantly overexpressed in the colon tissue of B27+ CHOP− compared to B27+ CHOP+ rats. Representative differences include pro-inflammatory genes like *Il1a*, *Tnf*, *Ifng*, *Nfkbiz* and *Mapk3* (Fig. 5C). *Il1b* expression trended higher in the absence of CHOP, but the difference was not significant. Three *Il17* isoforms (*a*, *c,* and *f*) were significantly increased in HLA-B27-Tg animals but were not significantly reduced by CHOP-deficiency. Interestingly, *Il17c* was significantly increased by eliminating CHOP expression in HLA-B27-Tg rats. Pathway analysis of the expression differences suggested an increase in IL-17 signaling in the absence of CHOP in HLA-B27-Tg colon tissue (Suppl. Fig. 4B) supporting the idea that CHOP protects against more severe gut inflammation. Other pro-inflammatory genes significantly elevated in HLA-B27+ CHOP− tissue compared with HLA-B27+ CHOP+ included *S100a9*, *Mmp9* and the chemokines *Cxcl11* and *Cxcl3* (Fig. 5C). As previously reported, *Apoa1* expression is reduced, and *Duox2* expression is elevated during gut inflammation^29^. This was also observed in HLA-B27-Tg inflamed colon tissue independently of CHOP expression with reduced *Apoa1* expression and increased expression of the antimicrobial *Duox2* and its adaptor *Duoxa2* (Fig. 5D). *Duoxa2* encodes for an endoplasmic reticulum protein mediating DUOX2 localization and maturation^30^, and was further significantly upregulated in the absence of CHOP in HLA-B27-Tg colon tissue. Overall, these data indicate that in the absence of CHOP key pro-inflammatory factors are further upregulated in HLA-B27-Tg rats and therefore CHOP might have a rather protective effect on HLA-B27 mediated gut inflammation.

## Discussion

In this study, we analyzed the role of CHOP in HLA-B27-mediated gut inflammation in experimental SpA. Our results clearly show that CHOP deficiency does not prevent gut inflammation in HLA-B27-Tg rats despite clear evidence that the absence of CHOP abrogates excess *Il23a* expression during UPR activation and results in significantly reduction of *Il23a* expression in immune cells isolated from inflamed gut tissue. Furthermore, *Il17a* expression in colon tissue and immune cells derived from the tissue was not decreased in the absence of CHOP, nor did in situ analysis using RNAScope reveal changes in the proportion of IL-17-producing T cells (*Cd3e* + /*Il17a* +). Taken together, these results demonstrate that HLA-B27 is not driving activation of the IL-23/IL-17 axis in the gut through UPR-mediated CHOP induction and increased *Il23a* expression as had been hypothesized.

Rather than promoting disease, CHOP appears to have a partially protective role in HLA-B27-induced colitis that becomes apparent at 6–7 months of age when inflammation is more severe^25^. This effect was uncovered when we compared colon histology scores from HLA-B27-expressing CHOP-deficient rats with HLA-B27-negative CHOP-deficient animals and arises because baseline colon histology scores in CHOP-deficient rats are lower than wild type animals. However, the increase in inflammation is not merely a consequence of changes in tissue cellularity because the expression of certain pro-inflammatory cytokines is also increased in HLA-B27+ rats lacking CHOP compared to either CHOP-deficient or wild type controls. Key pro-inflammatory cytokines such as TNF, IFNγ, and IL-1α were increased, as were chemokines that attract neutrophils (CXCL3 and CXCL11), S100A9, and the tissue degrading enzyme MMP9.

The partially protective role of CHOP in HLA-B27-Tg rats is in contrast to what has been shown for CHOP in other colitis models in mice. For example, dextran sodium sulfate (DSS)-induced colitis is suppressed in CHOP-deficient mice, due in part to the lack of CHOP-mediated apoptosis^31^. Consistent with this result, forced CHOP overexpression in intestinal epithelial cells (IECs) increased intestinal inflammation and mucosal tissue damage induced by DSS^32^. The tissue injury induced in CHOP overexpressing mice seemed to be independent of CHOP-mediated apoptosis but was associated with compromised IEC proliferation capacity and tissue repair. CHOP overexpression alone was not sufficient to induce intestinal inflammation in this model, indicating that additional stress signal was required. In another model examining a distinct signaling arm of the UPR, knocking out *Ire1a* (which senses ER stress and activates XBP1) in IECs resulted in spontaneous colitis^33^. Interestingly, in the absence of IRE1α, colonic ER stress was exacerbated in part by increased CHOP-induced apoptosis^33^. These contrasting roles of CHOP in DSS and *Ire1a*-deficient models compared to HLA-B27-induced colitis are likely a consequence of different disease mechanisms. For example, DSS causes epithelial damage, and the *Ire1a* knockout was IEC-specific, suggesting that they both directly impact epithelial barrier function exposing the underlying tissue to bacteria and pro-inflammatory contents of the intestine. In contrast, in experimental SpA HLA-B27 expression in bone marrow myeloid cells is sufficient to cause disease^34^, although damage to IECs, particularly when certain pathogens invade, could also influence severity^35^. Our results shown here in the HLA-B27 transgenic model emphasize that the pathogenesis of gut inflammation in HLA-B27 associated diseases may differ significantly from other types of inflammatory bowel disease where epithelial dysfunction is a key driver of the process. The availability of tools to create cell type-specific knockout rats would facilitate further experiments to address these questions.

We recently demonstrated that ERAP1-deficiency partially protects HLA-B27-Tg rats from developing arthritis but not gut inflammation^36^. In fact, colon histology scores were higher in the absence of ERAP1^36^, reminiscent of the effect of eliminating CHOP expression shown here. ERAP1-deficiency also reduced *Il23a* induction in myeloid cells by attenuating HLA-B27 misfolding and UPR activation^36^. We did not assess colon cytokine expression in the ERAP1 study. Nevertheless, these two studies are consistent and support the conclusion that CHOP induction, as a consequence of an HLA-B27 misfolding/ER stress-induced UPR, leading to IL-23 overexpression, is not driving gut inflammation in experimental SpA. It also seems unlikely that this mechanism is contributing to gut inflammation in HLA-B27-expressing humans, since UPR activation is not prominent in gut tissue^37^.

Defining the precise role of IL-23 in humans and animal models of SpA has been challenging. Genetic variants in the IL-23 receptor (*IL23R*) are involved in predisposition to ankylosing spondylitis^38^, and blocking IL-17A relieves symptoms^8^, while blockade of IL-23 or IL-12/23 through their common subunit has not proven beneficial^39^. In the ‘SKG’ mouse model where Th17 T cells accumulate due to a defect in negative selection, curdlan induces arthritis, enthesitis and ileitis, all of which are IL-23 dependent. In low copy number HLA-B27-Tg rats carrying extra hβ_2_m immunized heat inactivated mycobacterium tuberculosis and incomplete Freund’s adjuvant, arthritis can be prevented by blocking the IL-23R very early during the initiation phase of disease, but not after arthritis is established^40^. Gut inflammation in the low copy HLA-B27-Tg model is not a significant disease component, so effects of IL-23 in the gut are not known. It should be emphasized that our studies only address the role of ‘excess’ IL-23 produced from UPR-mediated CHOP induction, and thus determining whether non-UPR-mediated IL-23 plays a role in gut disease will require additional studies. It is possible that IL-23-independent IL-17A production from cells such as γδ T cells^28^ plays a role in the gut.

Significantly increased *Il17c* expression was apparent in CHOP-deficient HLA-B27+ colon tissue compared to B27+ CHOP+ . Different than the IL-17 isoforms A and F, IL-17C is produced mainly by epithelial cells and can act in synergy with TNF to induce pro-inflammatory cytokine production and immune cell infiltration into skin^41^, and thus could be contributing to the more severe inflammation seen in the absence of CHOP. In colonic epithelial cells, IL-17C is produced independent of IL-23^42^, and *Il17c* expression occurs before *Il17a* induction in DSS-induced colitis^43^. Interestingly, another gene whose expression was significantly increased in HLA-B27+ , CHOP-deficient rats is *Nfkbiz*, which encodes IKBζ. IKBζ is expressed in a variety of cells and together with RORyt can mediate the induction of Th17 cells in the absence of stimuli like IL-6 and TGFβ^44^. *NFKBIZ* expression is stimulated by TLR activation and cytokines like IL-17A and IL-1^45–47^. It is worth noting that stimulation of human synovial fibroblasts with TNF and IL-17A together resulted in synergistic increases in IKBζ leading to increased leukocyte recruitment through induction of chemokines and cytokines^48^. Thus, *NFKBIZ*/IKBζ may be an upstream mediator of increased chemokine and cytokine expression and exacerbating inflammation in HLA-B27-Tg, CHOP-deficient rats.

A limitation of our study is the inability to assess the role of CHOP in the development of HLA-B27-associated arthritis at this time. This phenotypic feature is uncommon when HLA-B27 is expressed in the Lewis strain in our animal facility. The lack of complete concordance between gut and joint inflammation in rats is similar to human SpA. As noted above, eliminating ERAP1 expression mitigates HLA-B27 misfolding and partially protects rats from the arthritis component of the SpA phenotype, while gut inflammation was exacerbated. Thus, it will be important to determine whether CHOP plays a role in arthritis. It is also unclear whether consequences of ER stress mediated through other arms of the UPR such as IRE1 and ATF6 play a role in HLA-B27-associated disease. Recent work identifying potential HLA-B27-binding peptides targeted by expanded clones of CD8+ T cells in patients with AS and acute anterior uveitis have rekindled interest in arthritogenic peptides^10^. However, links between cytotoxic T cells and activation of the IL-23/IL-17 axis activation remain unclear. Our data showing that UPR-driven, and CHOP-mediated excess IL-23 production does not drive colitis provide an important piece to the puzzle. Since CD8+ T cells are not driving disease in this animal model of SpA^4,5^, there may be other as yet unrecognized pathways independent of canonical ER stress signaling influenced by HLA-B27 expression that may contribute to pathogenesis.

In conclusion, CHOP deficiency does not prevent gut inflammation in HLA-B27-Tg animals, but rather it increases the severity of inflammation. Moreover, gut inflammation in this model is not dependent on CHOP and UPR-mediated increases in IL-23 production, indicating that other factors are driving the disease.

### Supplementary Information

Supplementary Information.

## Data Availability

The datasets generated and/or analyzed in this study will be made available from the corresponding author on reasonable request.
