# Supplementary material for: CHOP-mediated IL-23 overexpression does not drive colitis in experimental spondyloarthritis
Source: Sci Rep. 2024 May 29;14:12293. doi: 10.1038/s41598-024-62940-0 (PMC11137091; doi:10.1038/s41598-024-62940-0)
Supplement: Supplementary file 1 — Supplementary Information. [file 41598_2024_62940_MOESM1_ESM.docx]

**CHOP-mediated IL-23 overexpression does not drive colitis in experimental spondyloarthritis**

**Fatemeh Navid^1*^, Tejpal Gill^1^, Lilah Fones^1^, Jules D. Allbritton-King^2^, Kelly Zhou^1^, Isabel Shen^1^, Jinny Van Doorn^1^, Francesca LiCausi^1^, Antony Cougnoux^3^, Davide Randazzo^4^, Stephen R. Brooks^5^, Robert A. Colbert^1^**

**Supplemental Table I**

| **Genotype** | | **Name** |
| --- | --- | --- |
| *B27/hβ2m* | *Ddit3* |  |
| − | +/+ | B27-CHOP+  (Wildtype; WT) |
| + | +/+ | B27+CHOP+ |
| − | -/- | B27-CHOP- |
| + | -/- | B27+CHOP- |

CHOP-deficient (*Ddit3*-/-, CHOP-) Lewis rats were bred with HLA-B27-Tg (B27+CHOP+) rats to generate CHOP-deficient HLA-B27-Tg (B27+CHOP-) animals. Wild type (WT) (B27-CHOP+) and B27-CHOP- rats were used as controls.

**Supplemental Figure Legends**

**Supplemental Figure 1. Generation of *Ddit3*-/- rats.** (**A)** Coding sequence of *Ddit3* with 25 bp deletion (bold, underlined) created via CRSIPR/Cas9 editing. New in-frame stop codons are underlined. (**B**) Representative genotyping results for *Ddit3*+/+, *Ddit3*+/- and *Ddit3*-/- DNA showing the 25 bp deletion. (**C**) CHOP amino acid sequence (top) and predicted CHOP amino acid sequence (bottom) after 25 bp deletion leading to missense amino acids.

**Supplemental Figure 2. Increased HLA-B27 expression upon combined stimulation with proinflammatory cytokines.** (**A**) BMM from B27+CHOP+ rats were treated with IFNγ (I), TNF (T) or IFNγ+TNF (I+T) for 22 h or left untreated. Lysates were subjected to immunoblotting (IB) using HC10 (recognizes HLA-B27) and b-Tubulin antibody. Lines between lanes indicate splicing into a single figure. The images are all from the same immunoblot (original gel image is provided). Images are representative of three independent experiments. Blots were cropped to highlight the region of interest; full blot images are provided in Suppl. Fig. 5. (**B**) BMM from (**A**) were treated before lysis for 20 min on ice with MMTS. Lysates were used for immunoblotting in non-reducing condition using HC10 and b-Tubulin antibody. Blots were cropped to highlight the region of interest; full blot images are provided in Suppl. Fig. 6.

**Supplemental Figure 3. CHOP deficiency tends to affect differentially the distribution of B27+ histology scores.** Histological scores of 6-7 month old B27+CHOP+ and B27+CHOP- shown in Figure 3B were plotted showing the single categories: (**A**) goblet cell loss, (**B**) immune cell infiltrates, (**C**) area of tissue affected and (**D**) (GALT)/severity score.

**Supplemental Figure 4. CHOP-deficiency mediated expression differences in HLA-B27-Tg colon tissue.** (**A**) Clustering of expression data is based on predominantly on HLA-B27 expression and not CHOP deficiency. Heat map showing the unsupervised hierarchical clustering of expression data generated through RNASeq of colon samples from 7 WT, 7 B27-CHOP-, 10 B27+CHOP+ and 10 B27+CHOP- animals aged 6-7 mo. (**B**) TOPPGENE analysis of differentially expressed genes between B27+CHOP- and B27+CHOP+. On the y-axis are shown upregulated (red) and downregulated (blue) pathways and on the x-axis the -logP. (**D**) Expression of additional genes *Apoa1*, *Duox2*, and *Duoxa2*, with data derived and analyzed as in (**Fig. 4B**) (***p<* 0.01, ****p<*0.001).

Suppl. Figure 1

Suppl. Figure 2

Suppl. Figure 3

Suppl. Figure 4

Whole WB images Fig. 1A

Suppl. Figure 5

Whole WB images Suppl. Fig. 1B

Suppl. Figure 6

Whole WB images Suppl. Fig. 2

Suppl. Figure 7
